# Expression significance of Emi1, UBCH10 and CyclinB1 in esophageal squamous cell carcinoma

**DOI:** 10.3389/pore.2023.1611081

**Published:** 2023-04-24

**Authors:** Hui Li, Chenbo Yang, Kuisheng Chen, Miaomiao Sun

**Affiliations:** ^1^ Department of Pathology, The First Affiliated Hospital of Zhengzhou University, Zhengzhou, China; ^2^ Henan Key Laboratory of Tumor Pathology, Zhengzhou University, Zhengzhou, China

**Keywords:** tumor proliferation, Emi1, UBCH10, CyclinB1, tumor apoptosis

## Abstract

Despite significant advances in the diagnosis and treatment of esophageal squamous cell carcinoma (ESCC), esophageal cancer is still a heavy social and medical burden due to its high incidence. Uncontrolled division and proliferation is one of the characteristics of tumor cells, which will promote rapid tumor growth and metastasis. Early mitotic inhibitor 1 (Emi1), ubiquitin-conjugating enzyme 10 (UBCH10) and CyclinB1 are important proteins involved in the regulation of cell cycle. In this study, the expression of Emi1, UBCH10 and CyclinB1 in ESCC tissues and adjacent normal tissues will be analyzed by immunohistochemistry and *in-situ* hybridization techniques, and their relationship with tumor proliferation and apoptosis will be analyzed. The results showed that Emi1, UBCH10 and CyclinB1 genes and proteins were highly expressed in tumor tissues, which were correlated with tumor grade, lymph node metastasis and pathological stage, and positively correlated with tumor proliferation. Emi1, UBCH10 and CyclinB1 are also positively correlated. It is speculated that Emi1, UBCH10 and CyclinB1 genes synergically promote tumor proliferation and inhibit apoptosis, which may be potential diagnostic and therapeutic targets for ESCC.

## Introduction

Esophageal cancer is characterized by insidious onset, and patients often seek medical treatment in the middle and late stages, leading to poor prognosis and short survival time (1). Esophageal squamous cell carcinoma (ESCC) is a highly common subtype of esophageal carcinoma, so it is urgent to further explore the molecular pathology and the mechanism of malignant progression of ESCC. The uncontrolled proliferation of tumor cells is the result of dysregulation of cell cycle (2). Early mitotic inhibitor 1 (Emi1) mainly plays a role in promoting endogenous inhibitor of anaphase-promoting complex/cyclosome (APC/C), promoting the accumulation of S and G2 related mitotic Cyclin (3). Ubiquitin-conjugating enzyme 10 (UBCH10) is involved in the degradation of target proteins by the ubiquitin-proteasome system and thus plays a role in regulating the cell cycle (4). CyclinB1 belongs to the Cyclin family, which plays an important role in regulating the whole process of cell division and proliferation. Abnormal expression of cyclinB1 will cause the cell cycle to stall or exit, and the cell division and proliferation cannot be completed (5).

Currently, it is known that CyclinB1 is the target protein of ubiquitination protein degradation system, in which Emi1 and UBCH10 are participants. Studies have confirmed that Emi1, UBCH10 and CyclinB1 are abnormally expressed in a variety of tumors, and are closely related to the occurrence of tumors (6, 7). However, the expressions of Emi1, UBCH10 and CyclinB1 in ESCC, as well as whether there is interaction among them to jointly regulate the cell cycle process, are still unknown. Therefore, by exploring the expression of three proteins in ESCC and their correlation with tumor growth, we deeply understand the cell cycle process and regulatory mechanism of ESCC, hoping to provide a new theoretical basis for improving the therapeutic effect and prognosis of ESCC.

## Methods

### Tissue sample

All histological specimens were collected from confirmed ESCC and paracancer normal mucosal tissues from the Department of Pathology, the First Affiliated Hospital of Zhengzhou University from June 2020 to June 2021. All patients had not received radiotherapy or chemotherapy before surgery, and clinicopathologic data were complete. Among the 50 patients with ESCC, 29 were males and 21 were females. They ranged in age from 48 to 83, with a mean age of 60; 16 cases were classified as grade I-II and 34 cases as grade III. There were 23 cases with lymph node metastasis and 27 cases without lymph node metastasis. There were 27 cases of stage I to stage II and 23 cases of stage III to stage IV. Pathological staging was determined according to the TNM staging criteria for esophageal cancer (8th Edition) jointly published by the International Union Against Cancer (UICC) and the American Cancer Federation (AJCC) in 2017.

### Reagents

Rabbit anti-human Emi1 polyclonal antibody and rabbit anti-human UBCH10 polyclonal antibody were purchased from Proteintech, United States. Rabbit anti-human CyclinB1 monoclonal antibody was purchased from Shanghai Biyuntian Biotechnology Co., LTD. Rabbit anti-human Ki-67 monoclonal antibody was purchased from Shanghai Gene Technology Co., LTD. TUNEL test kit was purchased from Jiangsu Kaiji Biotechnology Co., LTD. *In situ* hybridization biotin labeled probe was designed and synthesized by Shanghai GenePharma Technology Co., LTD.

### Immunohistochemistry (IHC)

The slices were baked in a 61°C drying oven for 150min, and then dewaxed and hydrated. High pressure heating was used for antigen repair. Slices were added with appropriate 3% H_2_O_2_ drops, and then normal goat serum working solution was added. Add appropriate amount of primary antibody working solution and put the wet box in the refrigerator at 4°C overnight. On the second day, biotin labeled secondary antibody was added, then horseradish peroxidase labeled chain enzyme ovalbumin was added, and DAB working solution was added finally. ESCC tissue slices with known positive expressions of Emi1, UBCH10, CyclinB1 and Ki-67 were used as positive controls. PBS buffer will be used instead of primary antibody as the negative control.

Score by number of positive cells/percentage of observed cells. <1% is 0 point, 1%–25% is 1 point, 26%–50% is 2 point, 51%–75% is 3 point, >76% is 4 point. Score according to cell staining intensity: no color development is 0 point, light yellow is 1 point, brown and yellow is 2 point, tan is 3 point. Finally, the percentage score of positive cells was multiplied by the score of cell staining intensity to obtain the final score. The score ≤4 is negative, and the score >4 is positive.

### *In-situ* hybridization (ISH)

The *in-situ* hybridization biotin labeled probe sequence is shown below. Emi1 probe sequence: 5′-CAA​CTA​TCC​GAG​GGT​CGA​GG-3′. UBCH10 probe sequence: 5′-CAG​GGC​TCC​TGG​CTG​GTG​ACC​TGC​TT-3′. CyclinB1 probe sequence: 5′-CAG​TGA​CTT​CCC​GAC​CCA​GTA​GGT​ATT​T-3′.

The slices were baked in a 61°C drying oven for 150 min, and then dewaxed and hydrated. The slices were then dripped with an appropriate amount of 3% H_2_O_2_, followed by 0.3% Triton-X100. After pepsin was added, the pre-hybridization solution was added and incubated at 40°C for 3 h in a wet box. Biotin-labeled oligonucleotide probes were added and incubated overnight in a wet box at 42°C. On the second day, wash with SSC solution, add appropriate amount of sealing solution, and incubate in a wet box at 37°C for 30 min. Appropriate amount of SA-AP was added and incubated in a wet box at 37°C for 30 min. Appropriate amount of BCIP/NBT color developing working solution was added and incubated at 37°C in a wet box for 30–60 min. Appropriate amount of nuclear solid red dye solution was added to restain the nucleus for 5–15 min. ESCC slices with known positive expressions of Emi1, UBCH10 and CyclinB1 were used as positive controls. Hybridization solution without probe was used as negative control.

Score by number of positive cells/percentage of observed cells. <10% is 1 point, 11%–30% is 2 point, 31%–70% is 3 point, >70% is 4 point. Score according to cell staining intensity: no color development is 0 point, light blue is 1 point, darker purple blue is 2 point, deep purple blue is 3 point. Finally, the percentage score of positive cells was multiplied by the score of cell staining intensity to obtain the final score. The score <1 is negative, and the score ≥1 is positive.

### *In-situ* end labeling (TUNEL)

The slices were baked in a 61°C drying oven for 150 min, and then dewaxed and hydrated. Microwave heating was used to repair antigen. The slices was dripped with 3% H_2_O_2_, and then the sealer was dripped. Add TdT enzyme working solution and incubate at 37°C for 60 min in the dark. Streptavidin-HRP working solution was added and incubated at 37°C for 30 min in the dark. DAB working solution was added and color was developed for 3–7 min. The known positive tissue slices were treated with Dnase I as positive control. Reaction solution without TDT enzyme was used as negative control.

The degree of apoptosis was suggested by the apoptosis index (AI), which was calculated according to the number of positive cells/percentage of observed cells: AI = number of positive cells/200*100%.

### Statistical analysis

SPSS21.0 (United States) software was used for statistical analysis. Measurement data were expressed as ‾X ± S, and *t*-test was used to compare the differences between the two groups. Comparison of count data were performed by χ^2^ test or Fisher’s exact probability method, and correlation analysis was performed by Spearman method. Test level *α* = 0.05, *p* < 0.05 was considered statistically significant.

## Results

### The expression difference of Emi1, UBCH10 and CyclinB1 proteins in ESCC and paracancer tissues

**FIGURE 1:**
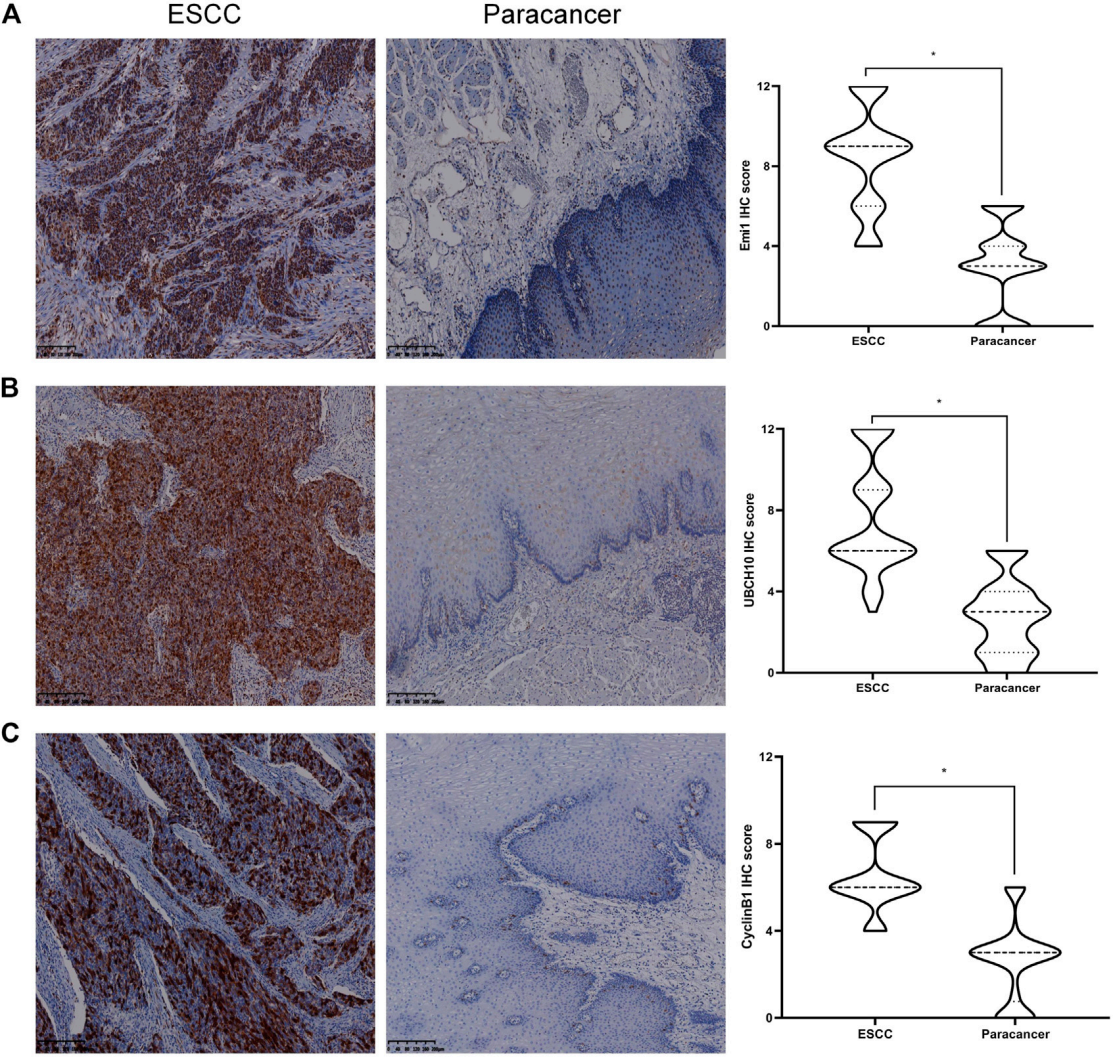
Expression of Emi1, UBCH10 and CyclinB1 proteins in ESCC and paracancer tissues. **(A)** Emi1 protein expression; **(B)** UBCH10 protein expression; **(C)** CyclinB1 protein expression (DAB color development, ×100).

The expressions of Emi1, UBCH10 and CyclinB1 proteins in ESCC and paracancer tissues were detected by IHC. The results showed that Emi1, UBCH10 and CyclinB1 proteins were highly expressed in ESCC tissues, as shown in Table 1 and Figure 1. There were significant differences in protein expression between ESCC and paracancer tissues (*p* < 0.05).

**TABLE 1:** The expression difference of Emi1, UBCH10 and CyclinB1 proteins in ESCC and paracancer tissues.

Group	Number of cases	Emi1				UBCH10				CyclinB1			
		+	−	χ^2^	*p*	+	−	χ^2^	p	+	−	χ^2^	*p*
ESCC	50	43	7	46.314	0.000*	44	6	54.782	0.000*	43	7	54.782	0.000*
Paracancer	50	9	41			7	43			6	44		

*p < 0.05.

### The expression difference of Emi1, UBCH10 and CyclinB1 mRNA in ESCC and paracancer tissues

**FIGURE 2:**
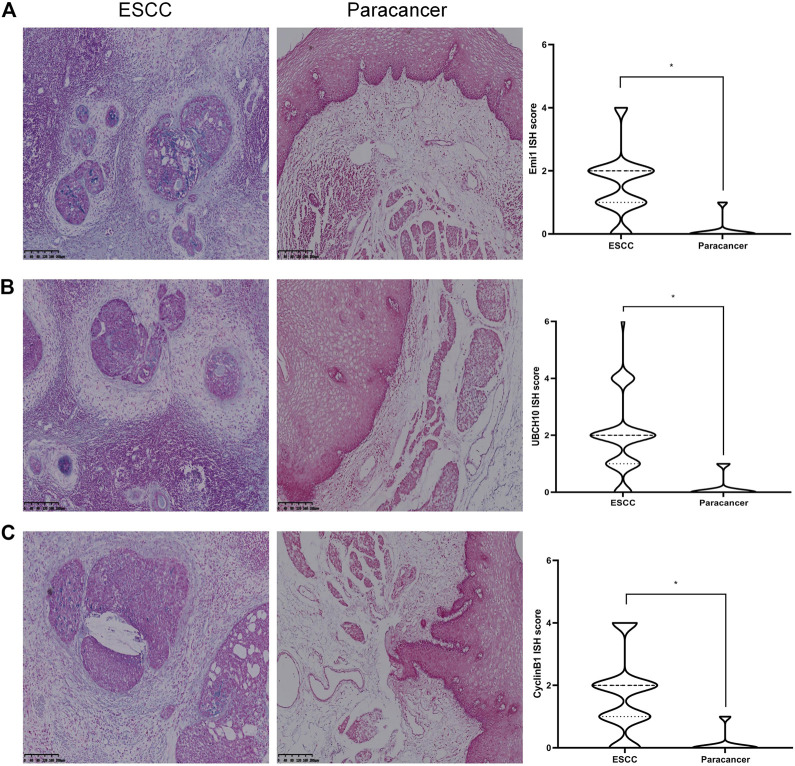
Expression of Emi1, UBCH10 and CyclinB1 mRNA in ESCC and paracancer tissues. **(A)** Emi1 mRNA expression; **(B)** UBCH10 mRNA expression; **(C)** CyclinB1 mRNA expression (BCIP/NBT color development, ×100).

The expressions of Emi1, UBCH10 and CyclinB1 mRNA in ESCC and paracancer tissues were detected by ISH. The results showed that Emi1, UBCH10 and CyclinB1 mRNA were highly expressed in ESCC tissues, as shown in Table 2 and Figure 2. mRNA expression was significantly different between ESCC and paracancer tissues (*p* < 0.05).

**TABLE 2:** The expression difference of Emi1, UBCH10 and CyclinB1 mRNA in ESCC and paracancer tissues.

Group	Number of cases	Emi1				UBCH10				CyclinB1			
		+	−	χ^2^	*p*	+	−	χ^2^	*p*	+	−	χ^2^	*p*
ESCC	50	43	7	54.782	0.000*	44	6	51.923	0.000*	41	9	46.314	0.000*
Paracancer	50	6	44			8	42			7	43		

*p < 0.05.

### Correlation between the expressions of Emi1, UBCH10, CyclinB1 and clinicopathological indexes

The correlation between Emi1, UBCH10, CyclinB1 and clinicopathological indexes was analyzed, including gender, age, tumor diameter, tissue grade, depth of invasion, lymph node metastasis, and pathological stage. The results showed that the expression of Emi1 protein and mRNA were correlated with tissue grade, lymph node metastasis and pathological stage (*p* < 0.05), as shown in Table 3. UBCH10 protein and mRNA expression were correlated with tissue grade, lymph node metastasis and pathological stage (*p* < 0.05), as shown in Table 4. CyclinB1 protein is correlated with tissue grade, lymph node metastasis, and pathological stage, while CyclinB1 mRNA is only correlated with tissue grade (*p* < 0.05). The difference between CyclinB1 protein and mRNA may be related to the small tissue sample size, as shown in Table 5.

**TABLE 3:** Correlation between the expressions of Emi1 and clinicopathological indexes.

Clinicopathological indexes	Number of cases	Emi1 protein				Emi1 mRNA			
		Positive (%)	Negative (%)	χ^2^	*p*	Positive (%)	Negative (%)	χ^2^	*p*
Gender									
Male	29	27 (93.10)	2 (6.90)	1.659	0.198	27 (93.10)	2 (6.90)	1.659	0.198
Female	21	16 (76.19)	5 (23.81)			16 (76.19)	5 (23.81)		
Age									
<60	17	16 (94.12)	1 (5.88)	0.573	0.449	14 (82.35)	3 (17.65)	0.011	0.918
≥60	33	27 (81.82)	6 (18.18)			29 (87.88)	4 (12.12)		
Tumor diameter (cm)									
<3	16	13 (81.25)	3 (18.75)	0.052	0.820	13 (81.25)	3 (18.75)	0.052	0.820
≥3	34	30 (88.24)	4 (11.76)			30 (88.24)	4 (11.76)		
Tissue grade									
G1–G2	16	11 (68.75)	5 (31.25)	3.899	0.048*	11 (68.75)	5 (31.25)	3.899	0.048*
G3	34	32 (94.12)	2 (5.88)			32 (94.12)	2 (5.88)		
Depth of invasion									
T1–T2	21	17 (80.95)	4 (19.05)	0.214	0.644	18 (85.71)	3 (14.29)	0.000	1.000
T3–T4	29	26 (89.66)	3 (10.34)			25 (86.21)	4 (13.79)		
Lymph node metastasis									
N^−^	27	20 (74.07)	7 (25.93)	6.934	0.011*	20 (74.07)	7 (25.93)	6.934	0.011*
N^+^	23	23 (100)	0 (0)			23 (100)	0 (0)		
Pathological stage									
I–II	27	20 (74.07)	7 (25.93)	6.934	0.011*	20 (74.07)	7 (25.93)	6.934	0.011*
III–IV	23	23 (100)	0 (0)			23 (100)	0 (0)		

N^+^, Lymph node metastasis; N^−^, No lymph node metastasis. *p < 0.05.

**TABLE 4:** Correlation between the expressions of UBCH10 and clinicopathological indexes.

Clinicopathological indexes	Number of cases	UBCH10 protein				UBCH10 mRNA			
		Positive (%)	Negative (%)	χ^2^	*p*	Positive (%)	Negative (%)	χ^2^	*p*
Gender									
Male	29	28 (96.55)	1 (3.45)	3.048	0.081	26 (89.66)	3 (10.34)	0.000	1.000
Female	21	16 (76.19)	5 (23.81)			18 (85.71)	3 (14.29)		
Age									
<60	17	15 (88.24)	2 (11.76)	0.001	1.000	15 (88.24)	2 (11.76)	0.000	1.000
≥60	33	29 (87.88)	4 (12.12)			29 (87.88)	4 (12.12)		
Tumor diameter (cm)									
<3	16	15 (93.75)	1 (6.25)	0.154	0.695	13 (81.25)	3 (18.75)	0.293	0.588
≥3	34	29 (85.29)	5 (14.71)			31 (91.18)	3 (8.82)		
Tissue grade									
G1–G2	16	11 (68.75)	5 (31.25)	5.794	0.016*	11 (68.75)	5 (31.25)	5.794	0.016*
G3	34	33 (97.06)	1 (2.94)			33 (97.06)	1 (2.94)		
Depth of invasion									
T1–T2	21	16 (76.19)	5 (23.81)	3.048	0.081	18 (85.71)	3 (14.29)	0.000	1.000
T3–T4	29	28 (96.55)	1 (3.45)			26 (89.66)	3 (10.34)		
Lymph node metastasis									
N^−^	27	21 (77.78)	6 (22.22)	5.808	0.025*	21 (77.78)	6 (22.22)	5.808	0.025*
N^+^	23	23 (100)	0 (0)			23 (100)	0 (0)		
Pathological stage									
I–II	27	21 (77.78)	6 (22.22)	5.808	0.025*	21 (77.78)	6 (22.22)	5.808	0.025*
III–IV	23	23 (100)	0 (0)			23 (100)	0 (0)		

N^+^, Lymph node metastasis; N^−^, No lymph node metastasis. *p < 0.05.

**TABLE 5:** Correlation between the expressions of CyclinB1 and clinicopathological indexes.

Clinicopathological indexes	Number of cases	CyclinB1 protein				CyclinB1 mRNA			
		Positive (%)	Negative (%)	χ^2^	*p*	Positive (%)	Negative (%)	χ^2^	*p*
Gender									
Male	29	27 (93.10)	2 (6.90)	1.659	0.198	26 (89.66)	3 (10.34)	1.646	0.200
Female	21	16 (76.19)	5 (23.81)			15 (71.43)	6 (28.57)		
Age									
<60	17	16 (94.12)	1 (5.88)	0.573	0.449	14 (82.35)	3 (17.65)	0.000	1.000
≥60	33	27 (81.82)	6 (18.18)			27 (81.82)	6 (18.18)		
Tumor diameter (cm)									
<3	16	14 (87.50)	2 (12.50)	0.000	1.000	12 (75.00)	4 (25.00)	0.239	0.625
≥3	34	29 (85.29)	5 (14.71)			29 (85.29)	5 (14.71)		
Tissue grade									
G1–G2	16	11 (68.75)	5 (31.25)	3.899	0.048*	9 (56.25)	7 (43.75)	8.160	0.004*
G3	34	32 (94.12)	2 (5.88)			32 (94.12)	2 (5.88)		
Depth of invasion									
T1–T2	21	16 (76.19)	5 (23.81)	1.659	0.198	17 (80.95)	4 (19.05)	0.000	1.000
T3–T4	29	27 (93.10)	2 (6.90)			24 (82.76)	5 (17.24)		
Lymph node metastasis									
N^−^	27	20 (74.07)	7 (25.93)	6.934	0.011*	19 (70.37)	8 (29.63)	3.802	0.051
N^+^	23	23 (100)	0 (0)			22 (95.65)	1 (4.35)		
Pathological stage									
I–II	27	20 (74.07)	7 (25.93)	6.934	0.011*	19 (70.37)	8 (29.63)	3.802	0.051
III–IV	23	23 (100)	0 (0)			22 (95.65)	1 (4.35)		

N^+^, Lymph node metastasis; N^−^, No lymph node metastasis. *p < 0.05.

### Correlation of Emi1, UBCH10 and CyclinB1 expression

**FIGURE 3:**
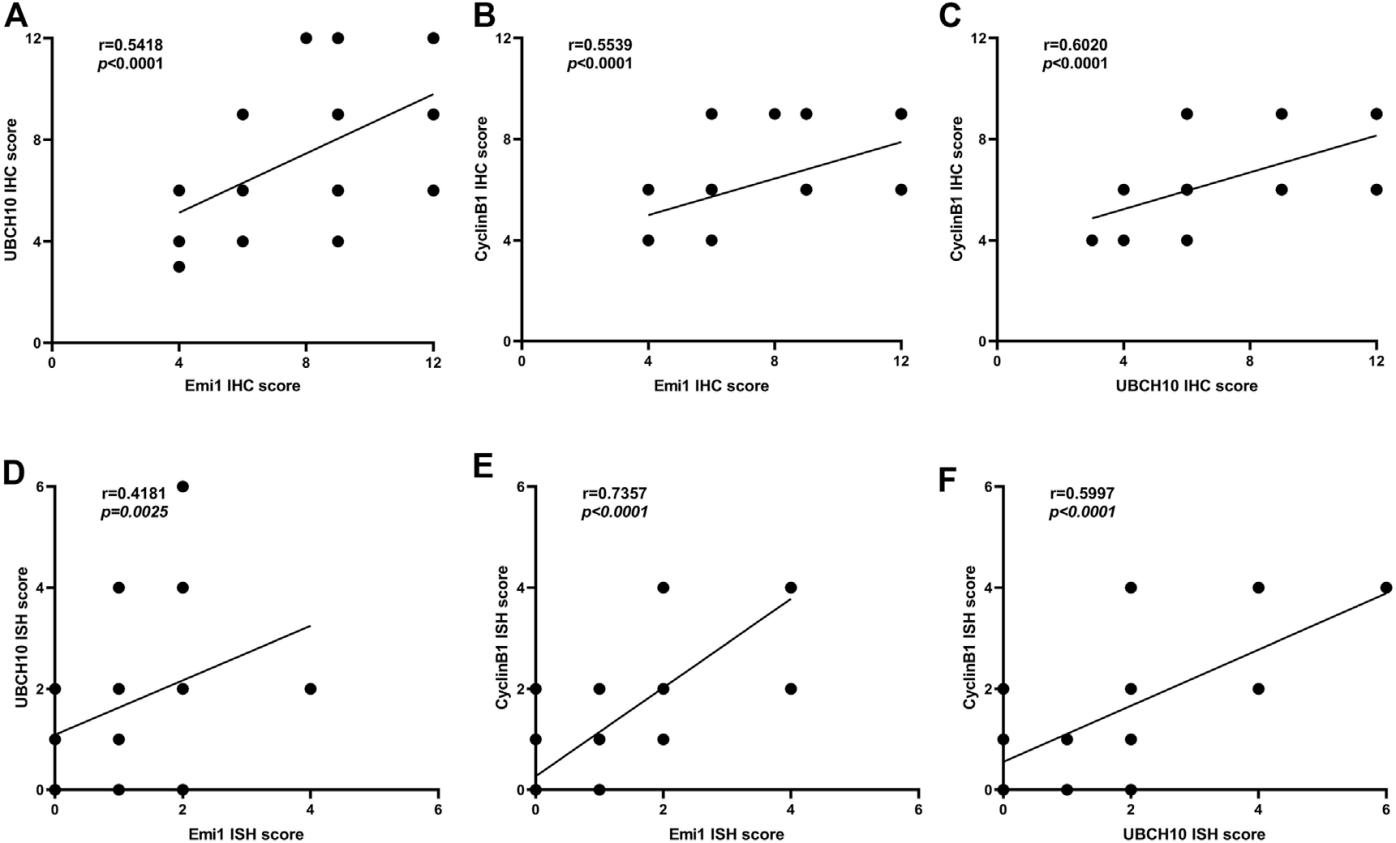
Correlation between Emi1, UBCH10 and CyclinB1 expression (A dot may represent multiple samples with the same score). **(A)** Protein expression between Emi1 and UBCH10; **(B)** Protein expression between Emi1 and CyclinB1; **(C)** Protein expression between UBCH10 and CyclinB1; **(D)** mRNA expression between Emi1 and UBCH10; **(E)** mRNA expression between Emi1 and CyclinB1; **(F)** mRNA expression between UBCH10 and CyclinB1.

The correlation of protein expression among Emi1, UBCH10 and CyclinB1 in ESCC tissues was analyzed, as shown in Figure 3. The results showed that Emi1 was positively correlated with UBCH10 protein expression (*r* = 0.5418, *p* < 0.0001). Emi1 was positively correlated with the expression of CyclinB1 (*r* = 0.5539, *p* < 0.0001). UBCH10 was positively correlated with the expression of CyclinB1 (*r* = 0.6020, *p* < 0.0001).

The mRNA expression correlation among Emi1, UBCH10 and CyclinB1 in ESCC tissues was further analyzed, as shown in Figure 3. The results showed that Emi1 was positively correlated with UBCH10 mRNA expression (*r* = 0.4181, *p* = 0.0025). Emi1 was positively correlated with the expression of CyclinB1 mRNA (*r* = 0.7357, *p* < 0.0001). UBCH10 was positively correlated with the expression of CyclinB1 mRNA (*r* = 0.5997, *p* < 0.0001).

### Proliferation and apoptosis in ESCC and paracancer tissues

**FIGURE 4:**
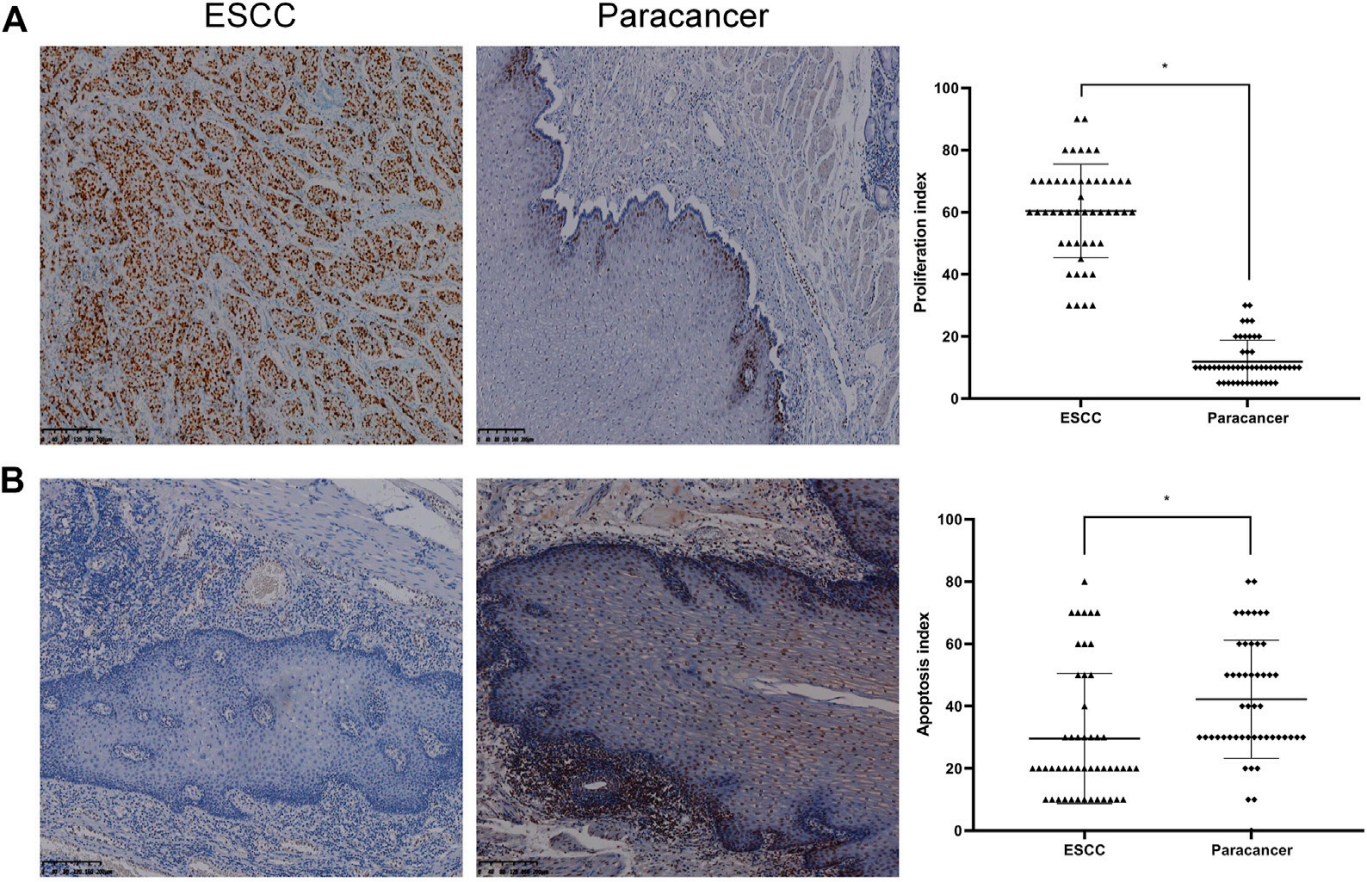
Proliferation and apoptosis in ESCC and paracancer tissues [**(A)** Proliferation in ESCC and paracancer tissue; **(B)** Apoptosis in ESCC and paracancer tissues] (DAB color development, ×100).

The expression of Ki-67 protein in ESCC and paracancer tissues was detected by IHC. Ki-67 is a nuclear antigen closely related to cell proliferation. The proliferation index was determined by evaluating the expression of Ki-67 in ESCC and paracancer tissues, as shown in Table 6 and Figure 4. The results showed that the ESCC tissue presented a very obvious high proliferation index, with a mean of 60.40%, while the proliferation index of the paracancer tissue was significantly reduced, about 11.90%, compared with the tumor tissue (*p* < 0.05).

**TABLE 6:** Proliferation index and apoptosis index in ESCC and paracancer tissue (‾X ± S) (%).

Group	Number of cases	Proliferation index		Apoptosis index	
		Mean ± s.d	*p*	Mean ± s.d	*p*
ESCC	50	60.40 ± 15.08	0.000*	29.60 ± 20.89	0.002*
Paracancer	50	11.90 ± 6.84		42.20 ± 18.98	

*p < 0.05.

Apoptosis in ESCC and paracancer tissues was determined by TUNEL technique, as shown in Table 6 and Figure 4. The results showed that the apoptotic index was about 29.60% in ESCC tissue and 42.20% in paracarcinoma tissue, and the apoptotic index was lower in ESCC tissue. The proliferation and apoptosis indexes of ESCC and paracancer tissues were significantly different (*p* < 0.05).

### Correlation between Emi1, UBCH10, CyclinB1 expression and tumor proliferation

**FIGURE 5:**
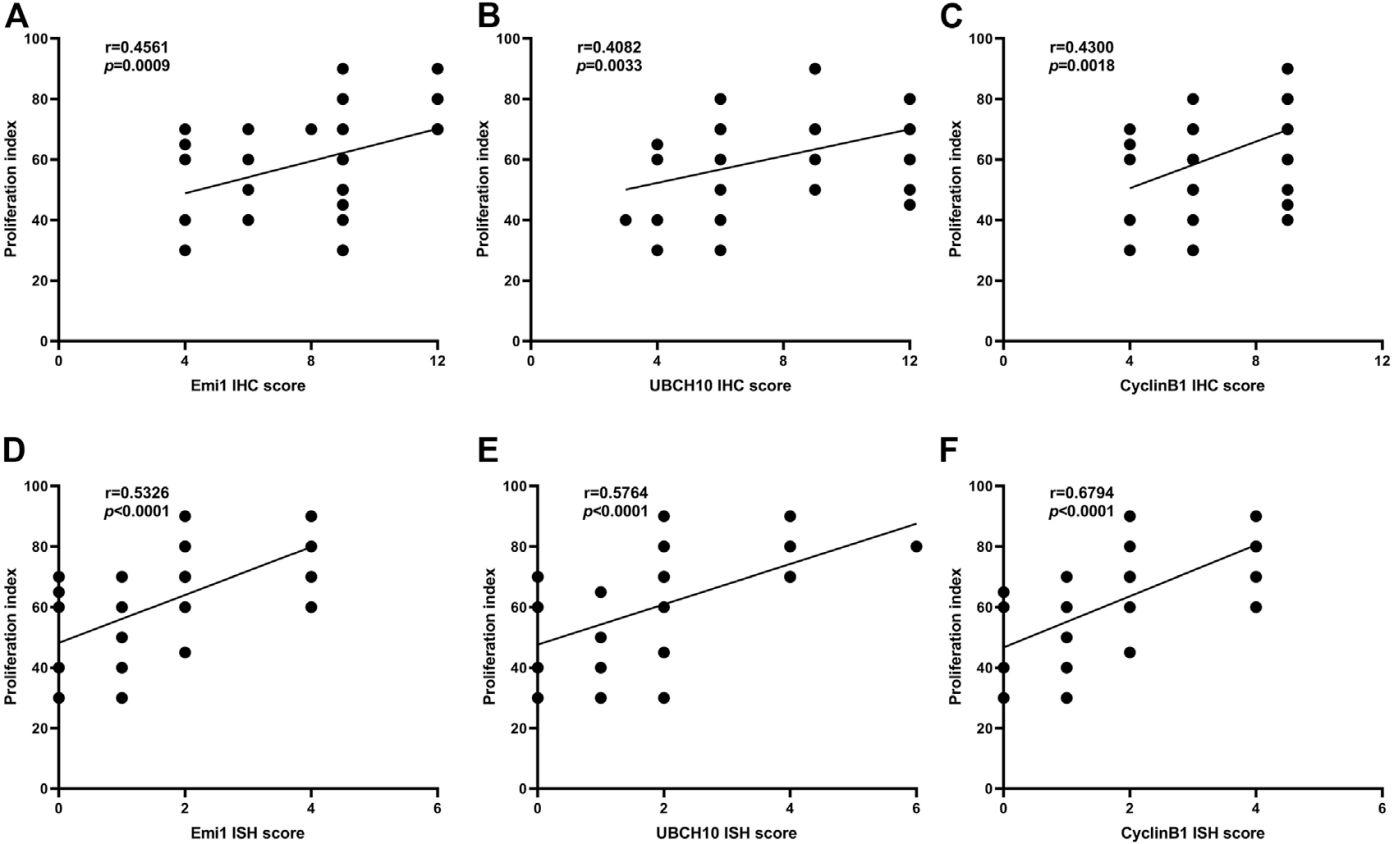
Correlation between Emi1, UBCH10, CyclinB1 and proliferation index (A dot may represent multiple samples with the same score). **(A)** Correlation between Emi1 protein and proliferation index; **(B)** Correlation between UBCH10 protein and proliferation index; **(C)** Correlation between CyclinB1 protein and proliferation index; **(D)** Correlation between Emi1 mRNA and proliferation index; **(E)** Correlation between UBCH10 mRNA and proliferation index; **(F)** Correlation between CyclinB1 mRNA and proliferation index.

The correlation between Emi1, UBCH10 and CyclinB1 protein expression and proliferation index in ESCC tissues was analyzed, as shown in Figure 5. The results showed that the protein expression of Emi1, UBCH10 and CyclinB1 was positively correlated with the proliferation index (*r* = 0.4561, *p* = 0.0009) (*r* = 0.4082, *p* = 0.0033) (*r* = 0.4300, *p* = 0.0018).

The correlation between Emi1, UBCH10 and CyclinB1 mRNA expression and proliferation index in ESCC tissues was analyzed, as shown in Figure 5. The results showed that the mRNA expression of Emi1, UBCH10 and CyclinB1 was positively correlated with the proliferation index (*r* = 0.5326, *p* < 0.0001) (*r* = 0.5764, *p* < 0.0001) (*r* = 0.6794, *p* < 0.0001).

### Correlation between expression of Emi1, UBCH10, CyclinB1 and tumor apoptosis

**FIGURE 6:**
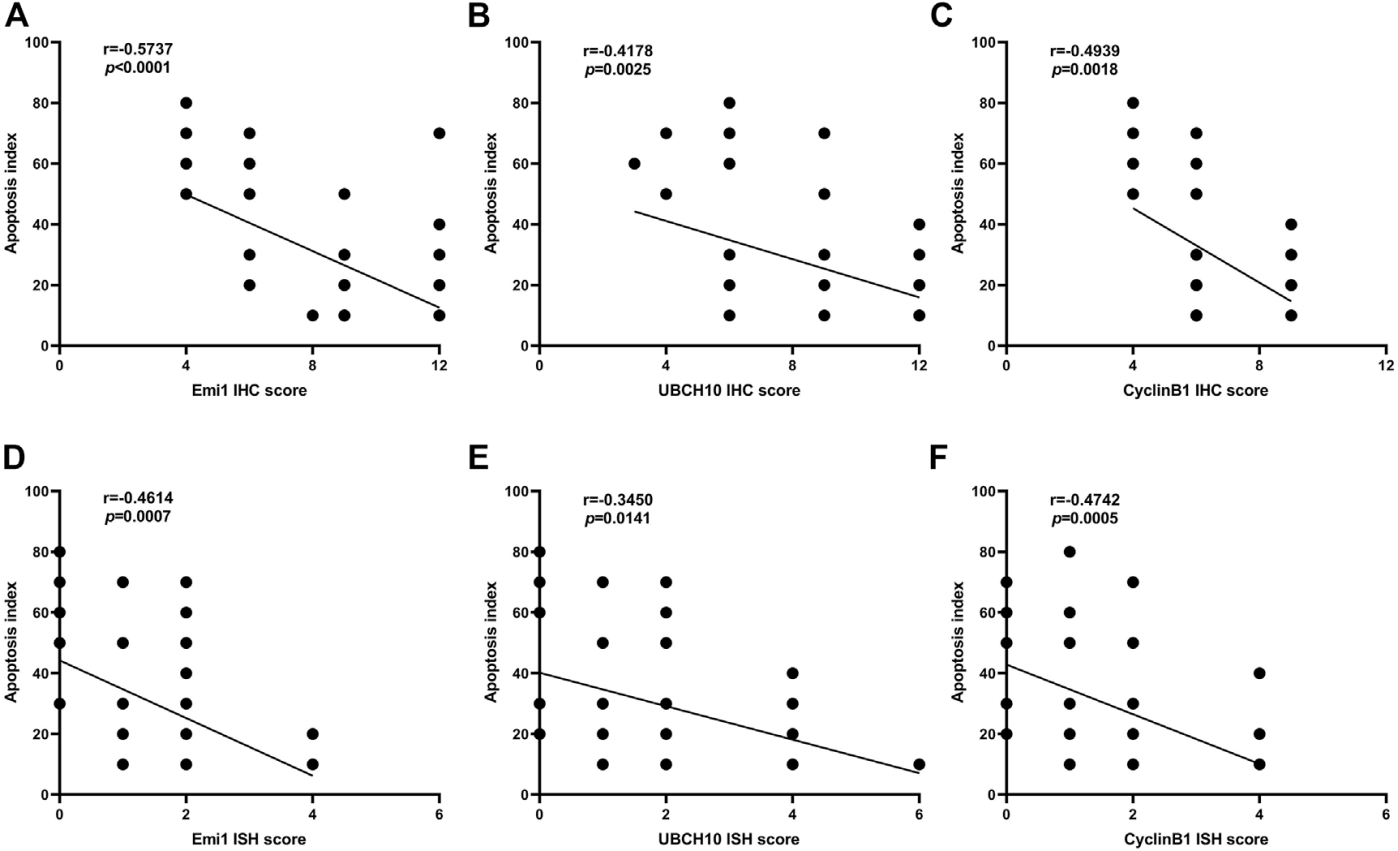
Correlation between Emi1, UBCH10, CyclinB1 and apoptosis index (A dot may represent multiple samples with the same score). **(A)** Correlation between Emi1 protein and apoptosis index; **(B)** Correlation between UBCH10 protein and apoptosis index; **(C)** Correlation between CyclinB1 protein and apoptosis index; **(D)** Correlation between Emi1 mRNA and apoptosis index; **(E)** Correlation between UBCH10 mRNA and apoptosis index; **(F)** Correlation between CyclinB1 mRNA and apoptosis index.

The correlation between Emi1, UBCH10, CyclinB1 protein expression and apoptosis index in ESCC tissues was analyzed, as shown in Figure 6. The results showed that the protein expressions of Emi1, UBCH10 and CyclinB1 were negatively correlated with the apoptosis index (*r* = −0.5737, *p* < 0.0001) (*r* = −0.4178, *p* = 0.0025) (*r* = −0.4939, *p* = 0.0018).

The correlation between Emi1, UBCH10 and CyclinB1 mRNA expression and apoptosis index in ESCC tissues was analyzed, as shown in Figure 6. The results showed that the mRNA expressions of Emi1, UBCH10 and CyclinB1 were negatively correlated with the apoptosis index (*r* = −0.4614, *p* = 0.0007) (*r* = −0.3450, *p* = 0.0141) (*r* = −0.4742, *p* = 0.0005).

## Discussion

Although a series of early screening work has been carried out in areas with high incidence of esophageal cancer, showing preliminary tumor prevention and control effect, the tumor burden of esophageal cancer is still very large. Finding new genes and proteins that may play an important role in the occurrence and development of ESCC and elucidating their related molecular mechanisms will be of great significance in the diagnosis and treatment of ESCC. Normal cells proliferate through mitosis and are regulated by a variety of cyclins, Cyclin-dependent protein kinases (CDKs), cell cycle checkpoints and cell cycle signaling pathways (8). The level of cyclin determines the mitotic process of cells. When the level of cyclin decreases, the cell cycle will actively stop or even exit the mitotic process. Protein ubiquitination plays an important role in the stabilization of cyclin levels (9).

Emi1 was initially thought to be a protein involved in the regulation of cell cycle. However, with the development of tumor research, researchers found that the “identity” of Emi1 in the development of tumor was actually oncogene (10). However, there are few reports on the expression of Emi1 in ESCC tissues, so this study started from exploring the expression of Emi1 gene in ESCC. We found that Emi1 mRNA and protein expressions in ESCC tumor tissues were higher than those in paracancer normal esophageal mucosa tissues, confirming the view that Emi1 is an oncogenic gene. Moreover, Emi1 expression was correlated with tumor differentiation, lymph node metastasis, and pathological stage, and was positively correlated with proliferation and negatively correlated with apoptosis, suggesting that Emi1 played an important role in the malignant process of ESCC. The findings of Emi1 in breast cancer are similar to our findings, Emi1 mediates the anti-apoptotic and pro-proliferative carcinogenicity of Skp2 through PI3K/Akt signaling pathway (11).

UBCH10 is an active protein in ubiquitin-proteasome. However, in addition to regulating ubiquitination degradation of protein, UBCH10 is also closely related to tumor proliferation and metastasis, and may be a new tumor marker or therapeutic target (4). Our study found that UBCH10 was significantly highly expressed in ESCC, which was related to tumor differentiation, lymph node metastasis, and pathological stage, and was positively correlated with tumor proliferation index and negatively correlated with apoptosis index, suggesting that UBCH10 could promote tumor proliferation and inhibit tumor apoptosis in ESCC. Compared with ESCC, UBCH10 has been extensively studied in other tumors. In glioma, UBCH10 expression increases with the increase of tumor malignancy. After siRNA silencing UBCH10, glioma cells show growth inhibition, cell cycle arrest and increased apoptosis (12, 13). UBCH10 in lung cancer is also related to tumor differentiation and patient survival, and UBCH10 can lead to P53 and EGFR gene mutations, resulting in loss of tumor inhibitory effect of P53 gene and enhancement of growth promoting effect of EGFR, while the proliferation of lung cancer cells and drug resistance of chemotherapy drugs are weakened after UBCH10 gene silencing (14, 15). UBCH10 is associated with ER and Ki-67 in breast cancer. Silencing UBCH10 can inhibit the proliferation of tumor cells and increase the sensitivity to chemotherapy. UBCH10 has also been detected in circulating tumor cells, suggesting that it can be used as an indicator for early screening and diagnosis of breast cancer (16).

CyclinB1 is a “star molecule” in the cyclin family and a core protein that regulates the G2 phase of the cell cycle. Similar to UBCH10, CyclinB1 not only has cell cycle regulation function, but also is an oncogenic gene associated with abnormal tumor proliferation. Through the detection of CyclinB1 mRNA and protein in ESCC tissues, it was found that the high expression of CyclinB1 in ESCC tissues was positively correlated with the proliferation index and negatively correlated with the apoptosis index. The expression of CyclinB1 protein was correlated with tumor grade, lymph node metastasis and pathological stage. Other researchers have found evidence that confirms our results, The expression of CyclinB1 was downregulated in cervical cancer cells, and it was found that tumor progression was inhibited and cell cycle was stagnated in G2/M phase, which ultimately delayed the process of tumor development (17, 18). When the level of CyclinB1 was reduced in breast cancer cells, the proliferation and migration of tumor cells were inhibited, and the cell cycle was stagnated in G2/M phase (7).

Emi1 is involved in cell cycle regulation by acting as an endogenous inhibitor of APC/C and hindering the degradation of its substrates by APC/C (19). UBCH10, as a member of ubiquitin binding enzyme E2 family, binds to ubiquitin ligase E3 to form a complex to initiate ubiquitin-proteasome degradation pathway, degrades APC/C substrate CyclinB, and finally plays a role in regulating cell cycle (20). CyclinB1 is mainly involved in the regulation of G2 phase of the cell cycle. In late mitosis, CyclinB1 combines with CDK1 to form a complex, Cyclinb1-CDK1, which phosphorylates APC/C and then promotes the degradation of CyclinB1 (21). Emi1, UBCH10 and CyclinB1 are all participants in the cell cycle regulation mechanism and are key points in the APC/C molecular mechanism network. By analyzing the correlation between the expressions of Emi1, UBCH10 and CyclinB1, the results confirmed that there was a positive correlation between Emi1, UBCH10 and CyclinB1 at both protein level and mRNA level. The degradation of cyclins is mainly dependent on ubiquitination, among which APC/C is an important ubiquitin-binding enzyme E3, and the degradation substrates of APC/C include a variety of proteins such as CyclinA and CyclinB. The silencing of Emi1 in cells will cause a large number of ubiquitination substrate proteins of APC/C, resulting in ubiquitination degradation of CyclinA and CyclinB, and insufficient accumulation of cyclin in cells, which cannot enter the next phase, and eventually lead to cell cycle arrest (22). However, overexpression of Emi1 in cells will block the catalytic site of APC/C and competitively prevent APC/C substrate proteins from binding to APC/C co-receptors, resulting in increased CyclinA and CyclinB levels and disorder of cell proliferation cycle (19). Normal cell cycle arrest and cell physiological apoptosis can not be carried out, which further leads to malignant cell proliferation, promotes cell proliferation and inhibits cell apoptosis. In-depth analysis of the inhibitory effect and mechanism of Emi1 revealed that Emi1 protein contains a variety of domains that block the substrate binding site of APC/C and inhibit the formation of ubiquitin chains (23). As ubiquitin binding enzyme E2, UBCH10 has the function of forming and extending ubiquitin chains, which can connect the substrate protein and ubiquitin ligase E3 through the ubiquitin chain, label ubiquitin on the substrate protein, and initiate the ubiquitination degradation process (24). In normal cells, Emi1 can effectively inhibit UBCH10 ubiquitin chain extension, thus effectively stabilizing the level of substrate protein (25).

At present, the specific molecular mechanism of Emi1, UBCH10 and CyclinB1 genes in promoting tumor proliferation and inhibiting apoptosis has not been reported. Combined with our experimental results, we conjectured that Emi1 reduced UBCH10 consumption by inhibiting ubiquitin chain extension between UBCH10 and CyclinB1, and inhibited the ubiquitination degradation of CyclinB1 protein, resulting in high expression of Emi1, UBCH10 and CyclinB1 in tumor tissues. Cell cycle regulation is a complex and huge mechanism network. In addition to the influence of UBCH10 and CyclinB1 proteins, Emi1 may also have other molecular effects, which requires further discussion.

## Data Availability

The original contributions presented in the study are included in the article/Supplementary Material, further inquiries can be directed to the corresponding authors.
