# Supplementary material for: Expression significance of Emi1, UBCH10 and CyclinB1 in esophageal squamous cell carcinoma
Source: Pathol Oncol Res. 2023 Apr 24;29:1611081. doi: 10.3389/pore.2023.1611081 (PMC10164988; doi:10.3389/pore.2023.1611081)
Supplement: Supplementary file 1 [file DataSheet2.PDF]

Number	Emi1 IHC		Emi1 ISH		UBCH10 IHC		UBCH10 ISH	
	ESCC	Paracancer	ESCC	Paracancer	ESCC	Paracancer	ESCC	Paracancer
1	9	6	2	0	12	6	2	1
2	4	0	0	0	6	1	0	0
3	6	0	1	0	9	1	4	1
4	4	3	0	0	6	3	0	0
5	9	0	1	0	6	0	1	0
6	6	3	2	0	6	4	2	0
7	6	4	2	0	9	3	2	0
8	9	3	1	0	6	1	1	0
9	9	4	1	0	6	0	1	0
10	9	4	2	0	9	4	2	0
11	9	3	1	0	9	1	1	0
12	4	3	0	0	6	3	0	0
13	12	3	2	0	12	3	2	0
14	6	0	1	0	6	0	1	0
15	9	6	1	0	6	3	1	0
16	9	0	2	0	6	0	2	0
17	6	3	0	0	6	3	2	0
18	9	4	1	0	12	4	2	0
19	9	3	4	0	6	1	2	1
20	6	4	1	0	4	1	1	0
21	9	6	2	0	12	3	2	0
22	9	3	1	0	6	0	1	0
23	9	0	2	0	6	0	0	0
24	6	0	2	0	9	4	4	0
25	4	3	1	0	4	3	2	0
26	12	3	2	1	6	1	4	0
27	9	6	2	0	6	1	2	1
28	9	3	1	0	6	1	2	0
29	9	6	4	1	9	6	2	0
30	9	3	2	0	6	3	4	0
31	9	4	2	0	12	4	2	0
32	9	3	1	0	6	2	2	0
33	9	3	2	0	6	1	2	0
34	8	6	2	1	12	6	4	1
35	6	3	1	0	6	2	0	0
36	6	0	1	0	6	3	1	0
37	4	3	1	0	4	3	1	0
38	9	3	1	0	12	4	1	0
39	9	0	0	0	4	2	2	0
40	12	4	2	0	12	3	4	0
41	12	6	4	0	12	4	2	1
42	4	3	0	0	3	2	0	0
43	12	6	2	1	12	6	6	1
44	12	0	2	0	6	2	2	0

45	12	4	2	0	12	6	2	0
46	12	0	2	0	9	3	2	0
47	9	4	2	1	9	6	4	0
48	4	0	0	0	4	3	1	0
49	12	4	2	0	9	4	4	0
50	12	6	4	1	9	6	2	1

CyclinB1 IHC		CyclinB1 ISH		ki-67 score		Tunel score	
ESCC	Paracancer	ESCC	Paracancer	ESCC	Paracancer	ESCC	Paracancer
9	3	2	1	45	10	20	30
4	0	0	0	30	5	60	60
6	0	2	0	70	10	20	30
6	3	0	0	60	20	70	70
6	0	0	0	40	5	20	40
6	3	1	0	60	10	60	70
6	3	1	0	60	10	50	50
6	0	1	0	50	5	30	50
6	3	1	0	30	10	20	60
6	3	1	0	60	10	20	40
6	3	1	0	50	5	20	20
4	3	1	0	70	20	80	70
9	1	2	0	80	5	30	50
9	0	1	0	40	5	20	30
6	3	1	0	50	10	10	70
6	1	2	0	70	5	30	50
6	3	0	0	60	25	30	60
9	3	1	0	60	10	10	30
6	3	4	1	80	10	10	30
4	2	0	0	40	5	70	70
6	3	2	0	60	20	20	60
6	0	1	0	50	5	10	30
6	0	2	0	70	10	20	30
6	3	2	0	70	10	20	50
4	3	0	0	60	10	70	80
9	6	2	0	70	10	20	30
6	3	4	0	60	15	10	10
6	3	2	0	60	20	10	80
6	6	2	1	60	10	10	30
9	3	4	0	80	10	20	30
6	3	2	0	70	10	10	30
6	3	1	0	30	15	10	40
6	3	2	0	60	10	20	50
9	6	2	1	70	25	10	40
9	3	1	0	60	10	30	20
6	0	1	0	50	10	20	30
4	3	0	0	30	5	50	50
9	3	1	0	50	10	20	30
6	0	2	0	60	5	50	0
9	3	4	0	80	20	40	30
9	3	4	0	70	20	20	30
4	3	0	0	40	5	60	60
9	6	4	1	80	30	10	10
6	0	2	0	70	10	10	20

6	3	2	0	70	15	10	30
6	0	2	0	70	10	70	50
9	6	2	1	90	30	30	30
4	0	0	0	65	5	70	70
6	4	2	0	70	10	30	50
9	6	4	1	90	25	20	30
