# Supplementary material for: Expression significance of Emi1, UBCH10 and CyclinB1 in esophageal squamous cell carcinoma
Source: Pathol Oncol Res. 2023 Apr 24;29:1611081. doi: 10.3389/pore.2023.1611081 (PMC10164988; doi:10.3389/pore.2023.1611081)
Supplement: Supplementary file 2 [file DataSheet1.PDF]

Number	Gender	Age	Diameter	Differentiatio	Lymph node metastasis	T stage	N stage	M stage
1	Male	53	5.8	G3	3 Groups	T3	N3	M0
2	Female	64	2.8	G2	No	T3	N0	M0
3	Female	67	2	G3	2 Groups	T3	N2	M0
4	Female	73	1.5	G2	No	T3	N0	M0
5	Male	56	2	G2	1 Group	T3	N1	M0
6	Male	69	4.5	G3	No	T1	N0	M0
7	Male	55	1.5	G3	No	T2	N0	M0
8	Female	66	4	G3	1 Group	T3	N1	M0
9	Male	66	2.5	G2	No	T3	N0	M0
10	Female	61	4.5	G3	No	T2	N0	M0
11	Female	70	2	G2	No	T2	N0	M0
12	Female	64	3	G3	No	T1	N0	M0
13	Male	64	6.5	G2	1 Group	T3	N1	M0
14	Female	49	2	G3	1 Group	T2	N1	M0
15	Male	83	3	G3	1 Group	T3	N1	M0
16	Female	62	4	G2	No	T3	N0	M0
17	Male	55	4.5	G3	No	T3	N0	M0
18	Male	70	6.5	G3	1 Group	T3	N1	M0
19	Female	71	3.5	G3	2 Groups	T3	N2	M0
20	Female	71	4	G3	No	T2	N0	M0
21	Male	63	7	G3	1 Group	T3	N1	M0
22	Male	56	2.8	G3	No	T3	N0	M0
23	Male	54	3	G2	No	T2	N0	M0
24	Female	71	3.5	G2	No	T3	N0	M0
25	Female	60	3	G3	No	T1	N0	M0
26	Male	58	3.2	G3	1 Group	T3	N1	M0
27	Male	63	6	G3	2 Groups	T3	N2	M0
28	Male	81	3	G3	No	T2	N0	M0
29	Male	75	2	G3	3 Groups	T3	N3	M0
30	Male	64	4.5	G3	No	T2	N0	M0
31	Male	57	4.5	G3	No	T3	N0	M0
32	Female	53	3	G2	2 Groups	T3	N2	M0
33	Male	70	5.5	G3	No	T3	N0	M0
34	Male	63	5.5	G3	3 Groups	T3	N3	M0
35	Male	58	6	G2	No	T3	N0	M0
36	Male	58	1.5	G2	1 Group	T3	N1	M0
37	Female	68	3.2	G2	No	T3	N0	M0
38	Male	71	1.8	G2	1 Group	T2	N1	M0
39	Female	57	3	G2	No	T2	N0	M0
40	Male	67	3.5	G3	No	T3	N0	M0
41	Female	64	3.5	G3	2 Groups	T2	N2	M0
42	Female	62	0.5	G2	No	T1	N0	M0
43	Male	61	3	G3	3 Groups	T3	N3	M0
44	Female	72	2.5	G3	1 Group	T2	N1	M0
45	Female	53	4	G3	1 Group	T3	N1	M0

46	Male	55	2.5	G3	No	T2	N0	M0
47	Female	62	2.3	G3	3 Groups	T2	N3	M0
48	Male	48	4	G3	No	T1	N0	M0
49	Male	50	4.5	G3	No	T2	N0	M0
50	Male	65	3.8	G3	3 Groups	T2	N3	M0

Pathological stage
Ⅳ
Ⅱ
Ⅲ
Ⅱ
Ⅲ
Ⅰ
Ⅱ
Ⅲ
Ⅱ
Ⅱ
Ⅱ
Ⅰ
Ⅲ
Ⅲ
Ⅲ
Ⅱ
Ⅱ
Ⅲ
Ⅲ
Ⅱ
Ⅲ
Ⅱ
Ⅱ
Ⅱ
Ⅰ
Ⅲ
Ⅲ
Ⅱ
Ⅳ
Ⅱ
Ⅱ
Ⅲ
Ⅱ
Ⅳ
Ⅱ
Ⅲ
Ⅱ
Ⅲ
Ⅱ
Ⅱ
Ⅲ
Ⅰ
Ⅳ
Ⅲ
Ⅲ

Ⅱ
Ⅲ
Ⅰ
Ⅱ
Ⅳ
